# School Closures and ED Visits for Suicidality in Youths Before and During the COVID-19 Pandemic

**DOI:** 10.1001/jamanetworkopen.2023.43001

**Published:** 2023-11-10

**Authors:** Yael Dvir, Clare Ryan, JungAe Lee

**Affiliations:** 1 Division of Child and Adolescent Psychiatry, Department of Psychiatry, University of Massachusetts Chan Medical School/UMass Memorial Medical Center, Worcester; 2 Department of Psychiatry, University of Massachusetts Chan Medical School/UMass Memorial Medical Center, Worcester; 3 Department of Population and Quantitative Health Sciences, University of Massachusetts Chan Medical School, Worcester

## Abstract

This cohort study investigates the association of COVID-19–related school closures with rates of emergency department suicidality visits among youths ages 12 to 25 years.

## Introduction

Emergency department (ED) visits for suspected suicide attempts among persons aged 12 to 25 years increased after May 2020.^1^ The public health response to the COVID-19 pandemic included widespread school closures.^2^ Schools offer well-documented support for youths and families.^2,3^ Describing the association between pandemic-related school closures and suicidality may inform future public health policy. Our study analyzed rates of youth ED suicidality visits (EDSVs), defined as visits for suicide and self-injury attempts, alongside patterns of school closure for Massachusetts and Texas.

## Methods

The UMass Chan Medical School institutional review board indicated that oversight and informed consent were not required for this cohort study because the study team did not access private identifiable information. This study followed the STROBE reporting guideline.

Massachusetts and Texas were compared because of differences in school closure patterns. Data examining kindergarten through grade 12 school operations in academic years 2020-2021 and 2021-2022 were publicly available through Burbio’s School Opening Tracker.^4^ We used in-person index (IPI), weighing virtual instruction at 0%, hybrid instruction (2-3 d/wk in person) at 50%, and traditional instruction (5 d/wk in person) at 100%. A higher value indicates more in-person education. The IPI was plotted by month from September 2020 to June 2022.

Rates of EDSVs in age groups 12 to 17 years and 18 to 25 years from March 2019 to September 2022 were acquired from the Massachusetts Department of Public Health and Texas Department of State Health Services. Given that school closures affected youths aged 12 to 17 years, those aged 18 to 25 years represented a control group. We adjusted raw data for state population and calculated monthly differences in EDSV rates between age groups. The autocorrelation function of the difference series over time exhibited characteristics of white noise, allowing for statistical tests with the assumption of independence. We used a 1-sample *t* test to examine differences in EDSV rates between age groups before and during the pandemic and a 2-sample *t* test to compare rates between states. A 2-sided test at a .05 level was used. Statistical analyses were conducted using R statistical software version 4.2.2 (R Project for Statistical Computing).

## Results

**Figure 1.**
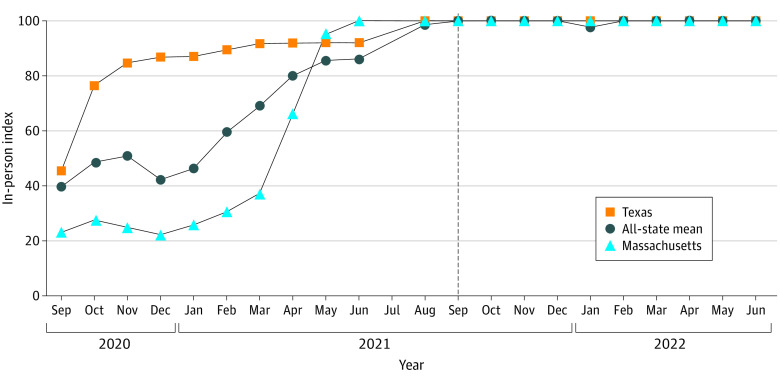
In-Person Index, 2020-2021 and 2021-2022 Academic Years Data provided by Burbio, Inc,^4^ 2023.

IPI plots (Figure 1) for Massachusetts and Texas show that Texas schools resumed in-person instruction with greater than 80% IPI beginning November 2020 while the Massachusetts IPI remained low (<40%) until May 2021. Both states are outliers compared with the all-state mean.

**Figure 2.**
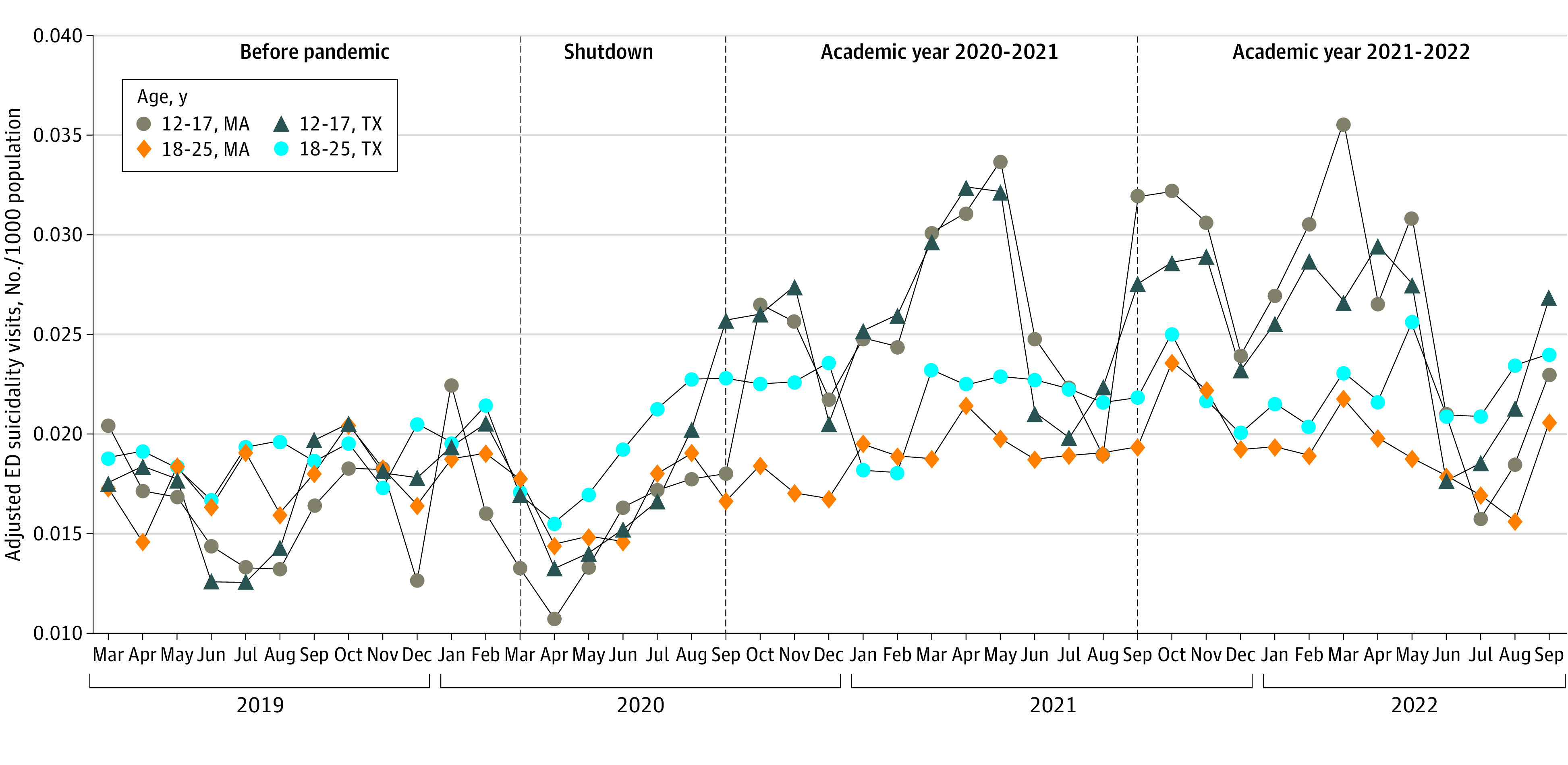
Emergency Department (ED) Visits for Suspected Suicide Attempts ED visits are shown among persons aged 12 to 25 years before and during the COVID-19 pandemic.

Before the pandemic (March 2019 to February 2020), mean (SD) monthly EDSV counts for individuals ages 12 to 27 years were 115 (21) visits in Massachusetts and 505 (82) visits in Texas. Between March and August 2020, schools were universally closed (school shutdown) and reopened starting September 2020. In academic year 2020-2021 and 2021-2022, mean (SD) counts were 176 (33) and 189 (43) visits, respectively, in Massachusetts and 756 (126) and 754 (122) visits, respectively, in Texas. Before the pandemic, there were no significant gaps by age group or state as confirmed by a 1-sample *t* test on the difference in EDSV rates between the 2 age groups in Massachusetts and Texas (Figure 2). Starting September 2020, youths aged 12 to 17 years had significantly higher rates of EDSVs in Massachusetts (*t* = 8.11; *df* = 23; *P* < .001) and Texas (*t* = 4.02; *df* = 23; *P* < .001) (Figure 2). Examining difference between states by a 2-sample *t* test revealed that the disparity between age groups 12 to 17 and 18 to 25 years in Massachusetts significantly expanded after September 2020 compared with Texas (*t* = 2.96; *df* = 46; *P* < .001) (Figure 2).

## Discussion

This cohort study found an association between longer school closures in the public health response to the COVID-19 pandemic and increases in youth suicidality. Limitations of the study include comparing only 2 states without considering other factors that may be associated with suicidality, which is outside the scope of this article. These data revealed an association between school closures and youth mental health, calling for further investigation such that in future pandemics and other disasters, policy regarding school closures may better align with the mental health needs of youths.

## Notes

Supplement.
Data Sharing Statement
