# Supplementary material for: School Closures and ED Visits for Suicidality in Youths Before and During the COVID-19 Pandemic
Source: JAMA Netw Open. 2023 Nov 10;6(11):e2343001. doi: 10.1001/jamanetworkopen.2023.43001 (PMC10638653; doi:10.1001/jamanetworkopen.2023.43001)
Supplement: Supplement. — Data Sharing Statement [file jamanetwopen-e2343001-s001.pdf]

Data Sharing Statement

Dvir. School Closures and ED Visits for Suicidality in Youths Before and During the COVID-19 Pandemic. *JAMA Netw Open*. Published online November 13, 2023. doi:10.1001/jamanetworkopen.2023.43001

Data
Data available: No

Additional Information
Explanation for why data not available: School closure data was publicly available through Burbio's School Opening Tracker. Emergency Department visit data is available upon request from from the MA Department of Public Health and the TX Department of State Health Services.
